# Effects of Tumor-Derived Exosome Programmed Death Ligand 1 on Tumor Immunity and Clinical Applications

**DOI:** 10.3389/fcell.2021.760211

**Published:** 2021-10-15

**Authors:** Bo Shao, Qin Dang, Zhuang Chen, Chen Chen, Quanbo Zhou, Bingbing Qiao, Jinbo Liu, Shengyun Hu, Guixian Wang, Weitang Yuan, Zhenqiang Sun

**Affiliations:** ^1^Department of Colorectal Surgery, The First Affiliated Hospital of Zhengzhou University, Zhengzhou, China; ^2^Academy of Medical Sciences, Zhengzhou University, Zhengzhou, China; ^3^School of Life Sciences, Zhengzhou University, Zhengzhou, China; ^4^Department of Hepatobiliary and Pancreatic Surgery, The First Affiliated Hospital of Zhengzhou University, Zhengzhou, China

**Keywords:** exosome, PD-L1, PD-1, tumor immunity, biomarker

## Abstract

Programmed death ligand 1 (PD-L1) is a typical immune surface protein that binds to programmed cell death 1 (PD-1) on T cells through its extracellular domain. Subsequently, T cell activity is inhibited, and tumor immune tolerance is enhanced. Anti-PD-1/PD-L1 immune checkpoint therapy blocks the combination of PD-1/PD-L1 and rejuvenates depleted T cells, thereby inhibiting tumor growth. Exosomes are biologically active lipid bilayer nanovesicles secreted by various cell types, which mediate signal communication between cells. Studies have shown that PD-L1 can not only be expressed on the surface of tumor cells, immune cells, and other cells in the tumor microenvironment, but also be released from tumor cells and exist in an extracellular form. In particular, exosome PD-L1 plays an unfavorable role in tumor immunosuppression. The immunomodulatory effect of exosome PD-L1 and its potential in fluid diagnosis have attracted our attention. This review aims to summarize the available evidence regarding the biological characteristics of exosome PD-L1 in tumor immunity, with a particular focus on the mechanisms in different cancers and clinical prospects. In addition, we also summarized the current possible and effective detection methods for exosome PD-L1 and proposed that exosome PD-L1 has the potential to become a target for overcoming anti-PD-1/PD-L1 antibody treatment resistance.

## Introduction

Programmed cell death-1 (PD-1), also known as CD279, is expressed in a variety of immune cells, including peripheral activated T cells, B cells, and monocytes (Nishimura et al., 1996; Keir et al., 2008). The two known ligands of PD-1 are Programmed death-ligand 1 PD-L1 (B7-H1) and PD-L2 (B7-DC) (Blank et al., 2004). PD-L1 is a typical immune surface protein that binds to PD-1 on T cells through its extracellular domain (Dong et al., 1999). PD-L1 inhibits the activity of T cells and enhances the immune tolerance of tumor cells, thereby preventing the immune response, which may damage the tumor, and leading to the immune escape of the tumor. PD-L1 was the first PD-1 ligand to be discovered. Numerous studies have shown that PD-L1 is abnormally expressed in many tumors, such as skin, brain, thyroid, esophageal, and colorectal tumors (Lin et al., 2015; Wang et al., 2016; Zhou et al., 2017). Therefore, PD-L1 is considered to be a critical factor involved in tumor immune escape.

Exosomes are biologically active extracellular vesicles approximately 30–120 nm in diameter with a lipid bilayer structure that are secreted by various cells (Théry et al., 2002). Exosomes are intraluminal vesicles (ILVs) formed by inward endosomal membrane budding during the maturation of multiple vesicular endosomes (MVEs; Colombo et al., 2014; Hessvik and Llorente, 2018). MVEs can fuse with lysosomes, leading to the degradation of extracellular vesicles and the recycling of their contents, which promotes cellular metabolism. MVEs also fuse with the cell membrane, causing ILVs to be released extracellularly, and these ILVs that are released from the cell are called exosomes (Colombo et al., 2014). Exosomes are released by various cell types and are stably present in all body fluids (Boukouris and Mathivanan, 2015). It has been widely validated that exosome facilitate communication between cells and the exchange of proteins, nucleic acids, and other substances (Lo Cicero et al., 2015; Cordonnier et al., 2017; Mashouri et al., 2019). Exosomes precisely transfer many biological components to target cells and are an effective way to affect gene expression in distant cells. These biological components are wrapped in a double membrane, which is stable even after being transferred to a remote location (Kourembanas, 2015; van Niel et al., 2018). Exosome biogenesis is a complex process. Lipid- and membrane-associated proteins accumulate in discrete membrane microdomains of the MVE, which then recruits soluble components such as cytoplasmic proteins, RNA, DNA, and cytokines (Nolte-’t Hoen et al., 2012; Villarroya-Beltri et al., 2013; Thakur et al., 2014). This process in turn involves important subunits of the endosomal complex required for transportation (ESCRT). Various subunits of ESCRT are involved in the formation of the entire ILV (Colombo et al., 2013). Recent experimental data indicate that interfering with the RNA of genes associated with ESCRT inactivating these proteins and components affect the secretion efficiency and composition of ILVs (Monypenny et al., 2018). However, some researchers have found that after knocking out the ESCRT complex, exosomes containing the marker CD63 are still present, which means that there may be ESCRT-independent ways to produce exosomes (Stuffers et al., 2009). Exosomes are involved in a wide range of processes, such as metabolic reprogramming (Thakur et al., 2020), macrophage M2 polarization (Ono et al., 2020), tissue repair (Roefs et al., 2020), osteogenic differentiation (Colletti et al., 2020), and hair regeneration (Hu et al., 2020). Emerging evidence suggests that tumor cells attenuate anti-tumor immunity by expressing biologically active PD-L1 on the surface of their secreted exosomes. In this review, we mainly summarize the mechanism of tumor-derived exosome PD-L1 in the context of tumor immunity and its potential significance in distinct tumor types.

## Biogenesis and Molecular Characteristics of Exosome PD-L1

Programmed death ligand 1 is a protein that is expressed on the cell surface. Activation of the PD-1/PD-L1 pathway mainly leads to tumor immune escape and promotes tumor cell growth by affecting T cell tolerance, T cell apoptosis, and T cell failure and enhancing Treg cell functions (Ichikawa and Chen, 2005; Mittal et al., 2014; Zhang et al., 2015). PD-L1 is expressed on the surface of tumor cells (Wang et al., 2016) and promotes tumor immune escape (Daassi et al., 2020). However, the tumor immune escape mediated by PD-L1 on the cell surface is temporary and dependent on IFN-γ. Moreover, the expression time of PD-L1 on the surface of tumor cells is extremely short. PD-L1 is also expressed on the surface of host immune cells, especially tumor-associated macrophages (TAMs). While PD-L1 disappears from the surface of tumor cells, PD-L1 expression on host immune cells is maintained (Noguchi et al., 2017). Therefore, although PD-L1 on the surface of tumor cells plays a certain role in immune escape, the establishment of an immunosuppressive tumor microenvironment is mainly realized by the expression of PD-L1 on the surface of TAMs.

**FIGURE 1:**
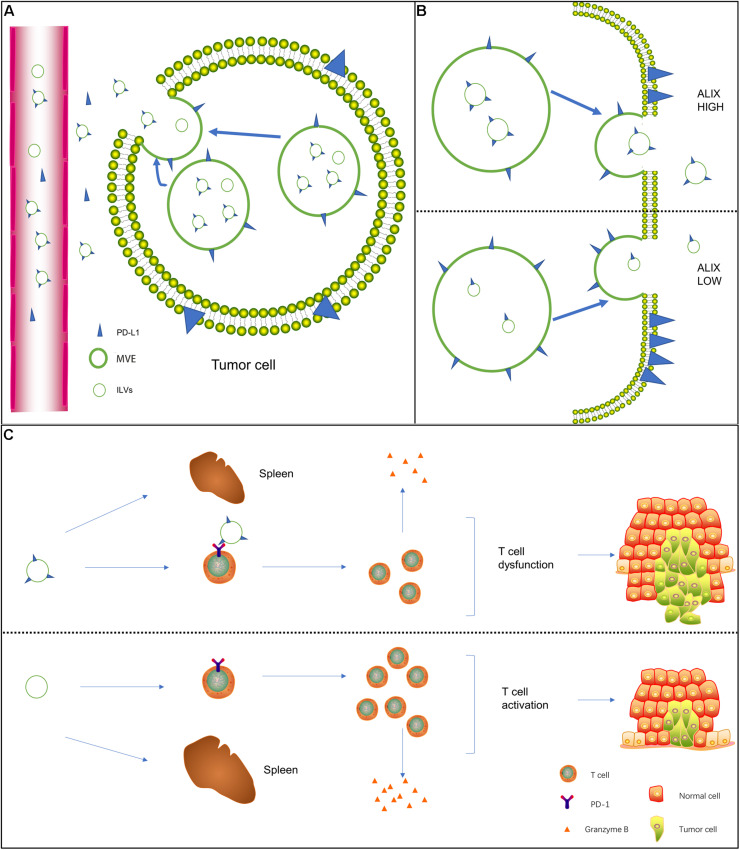
Biogenesis of exosome PD-L1 and its mechanism of action on T cells. **(A)** In addition to being expressed on the cell surface, free PD-L1 is released from tumor cells to the extracellular space. Free PD-L1 can be divided into exosome PD-L1 and soluble PD-L1. **(B)** The negative EGFR regulator protein ALIX seems to regulate the assembly of certain exosome cargoes. In ALIX-knockdown cells, PD-L1 expression on the cell surface was strongly increased, but PD-L1 significantly entered exosomes. This decrease indicates that ALIX controls the balance between exosome PD-L1 and cell surface PD-L1. **(C)** In the presence of exosome PD-L1, the number of activated T cells decreased, the activity of these cells was significantly reduced, the spleen was reduced in size, the level of granzyme B secreted by T cells was significantly reduced, the killing ability of T cells was inhibited, and tumor growth was significantly promoted.

In addition to being expressed on the cell surface, PD-L1 is also released from tumor cells into the extracellular space to become free PD-L1, including exosome PD-L1 and soluble PD-L1 (sPD-L1; Frigola et al., 2011; Theodoraki et al., 2018). However, before the study of exosome PD-L1 received attention, the study of extracellular PD-L1 mainly focused on the effect of sPD-L1 on cancer (Xing et al., 2012; Shi et al., 2013; Nagato et al., 2017). Moreover, the total amount of PD-L1 in circulation did not distinguish between soluble and exosome forms. Recent studies have shown that exosome PD-L1 plays an important role in tumor immunosuppression (Ludwig et al., 2017; Chen et al., 2018; Ricklefs et al., 2018). Compared with sPD-L1, exosome PD-L1 is not easily degraded by extracellular proteolytic enzymes and can induce T cell dysfunction and improve stability (Fan et al., 2019). Therefore, it is important to understand how PD-L1 on cells is assembled into exosomes. Monypenny et al. (2018) showed that ALIX, a negative regulator of EGFR, regulates the assembly of certain exosome cargo and controls the balance between exosome PD-L1 and cell surface PD-L1. In ALIX-knockdown cells, the proportion of PD-L1 present in the MVEs was larger than that in ILVs, and MVEs were easily observed in the budded state, indicating that ALIX is required for the processing of PD-L1 from the MVE membrane into the ILV (Figures 1A,B). Cordonnier et al. (2020) showed that sPD-L1 may not be a reliable biomarker for melanoma compared to exosome PD-L1. In addition, macrophages and dendritic cells (DCs) release exosomes containing PD-L1 (Hong et al., 2016; Ricklefs et al., 2018; Cordonnier et al., 2020). Interestingly, exosomes in the plasma of patients with chronic lymphocytic leukemia were found to be rich in non-coding Y RNA hY4 (Haderk et al., 2017). Additionally, transfer of CLL derived exosomes or hY4 alone to monocytes could lead to key CLL-associated phenotypes, which includes the release of cytokines as well as PD-L1 expression. In sum, the connection between tumor or immune cells and exosome PD-L1 may be highly intricate.

## Tumor-Derived Exosomes PD-L1 Can Regulate Immune Cells to Promote Tumor Growth

### Effect of Exosome PD-L1 on T Cells

Programmed death ligand 1 on the cell surface facilitates tumor immune escape by inducing activated T cell apoptosis, promoting T cell weakness, enhancing the function of regulatory T (Treg) cells, inhibiting T cell proliferation, activating damaged T cells, and stimulating the production of IL-2 (Zhang et al., 2015; Sun et al., 2018). Accumulating evidence has shown that exosome PD-L1 also plays a role in tumor immune escape and promotes tumor development by promoting T cell apoptosis and inhibiting the production of cytokines (Chen et al., 2018; Guo et al., 2019). Similarly, Kim et al. (2019) showed that exosomes containing PD-L1 could be isolated from the plasma of non-small cell lung cancer (NSCLC) patients. Tumor cell-derived exosome PD-L1 interacts with the PD-1 receptor on CD8 T cells, weakening the function of CD8 T cells, inducing their apoptosis, and promoting tumor immune escape (Kim et al., 2019). Therefore, membrane proteins on the surface of exosomes perform functions in a variety of tumors through direct protein-protein interactions (Table 1).

**TABLE 1:** Tumor-derived exosome PD-L1 in cancers.

**Tumor types**	**Target cell**	**Mechanisms**	**Tumor progression**	**References**
NSCLC	T cell	Exosome PD-L1 inhibits cytokines (IL-2 and IFN-γ) and induces apoptosis of CD8^+^ T-cells	↑	Kim et al., 2019
Melanoma	T cell	Exosome PD-L1 pass through T lymphocytes of secondary lymphoid organs and play a role through the immunosuppressive pathway of PD-1/PD-L1	↑	Cordonnier et al., 2020
Gastric cancer	T cell	Reduces the expression of CD69 and PD-1 on the surface of T cells, resulting in T cell dysfunction	↑	Fan et al., 2019
Breast cancer	T cell	Blocks phosphorylation of src family proteins, LAT and PLCγ in CD8 T cells, and promotes CD8 T cell dysfunction	↑	Chatterjee et al., 2020
HNSCC	T cell	Exosome PD-L1 inhibits and interferes CD8^+^ effector T cell activation	↑	Theodoraki et al., 2018

*↑, tumor promotion; NSCLC, non-small cell lung cancer; HNSCC, head and neck squamous cell carcinoma.*

Almost all previous studies on the interaction between PD-L1 and T cells were based on the surface PD-L1-mediated immunosuppression model of tumor cells (Iwai et al., 2002; Mittendorf et al., 2014). Whether exosome PD-L1 binds to PD-1 on T cells and inhibits the activity of CD8 T cells remains unknown. Yang et al. (2018) found that exosome PD-L1 was located on the surface of target cells and bound to PD-1, indicating that exosomes transfer functional PD-L1 to other cells. PD-1 and PD-L1 are part of the exosome cargo and modify the surface of exosomes in the serum of head and neck squamous cell carcinoma (HNSCC) patients (Theodoraki et al., 2019). Poggio et al. (2019) showed that in the presence of exosome PD-L1, T cells in tumor-draining lymph nodes expressed exhaustion markers, and the spleen was reduced in size (Figure 1C). The activation, proliferation, and killing potential of T cells is significantly enhanced by the removal of exosomes at the genetic level or by the deletion of PD-L1. When exogenous exosome PD-L1 was reintroduced, the effect was reversed (Poggio et al., 2019). Chen et al. (2018) established a mouse melanoma model with knockout of PD-L1 expression in B16-f10 cells [PD-L1 (KD) B16-F10 cells]. After the injection of exosomes derived from parental B16-F10 cells, the growth of tumors derived from PD-L1 (KD) B16-F10 cells was promoted, and the number of CD8 T lymphocytes invading the tumor was downregulated (Chen et al., 2018). The growth of tumor cells stimulated with exosomes containing PD-L1 was considerably increased compared with that of the control group in a constructed mouse breast cancer model (Yang et al., 2018).

### Exosome PD-L1 Can Promote Tumor Immune Escape by Inducing Macrophage M2 Polarization

Macrophages are derived from monocytes, which in turn are derived from precursor cells in the bone marrow. Macrophages are usually used to maintain the homeostasis of the internal environment and resist the invasion of pathogens (Davies et al., 2013). Macrophages in different environments will produce corresponding polarization, such as common M1 macrophages and M2 macrophages (Martinez and Gordon, 2014). M1 macrophages can promote inflammation and release pro-inflammatory related factors, while M2 macrophages can resist inflammation, play important roles in immunity, tissue homeostasis, metabolism, and endocrine signal transduction, and can promote tumor metastasis and proliferation (Funes et al., 2018).

Previous studies have found that tumor-derived exosomes can induce M2 polarization of macrophages. For example, studies by Gabrusiewicz et al. (2018) have shown that exosomes derived from glioblastoma stem cells can pass through the monocyte cytoplasm and cause muscle activity. The recombination of protein skeleton transformed monocytes into immunosuppressive M2 type, and the expression of PD-L1 in macrophages increased. Haderk et al. (2017) found that when exosomes from chronic lymphocytic leukemia transfer to monocytes, they will cause inflammation, lead to cancer, and increase the expression of PD-L1, and make tumor immune escape. According to the above research, the focus of previous researchers is the change of PD-L1 in M2 macrophages and the impact on tumors. However, there is little research on whether PD-L1 in tumor-derived exosomes influences macrophage polarization. Tumor cells increase the release of glutamate through the cystine/glutamate transporter cystine-glutamate exchange (xCT) to balance the oxidation homeostasis in tumor cells and promote tumor progression (Okazaki et al., 2017). The latest study by Liu et al. (2021) found that inhibiting xCT in melanoma can cause the transcription factor IRF4/EGR1 to upregulate the expression of PD-L1, which leads to melanoma cells secreting many exosomes carrying PD-L1, which in turn induces M2 macrophages polarized and reduced the efficacy of anti-PD-1/PD-L1 in the treatment of melanoma. And it was further discovered that sulfasalazine (SAS) induced macrophage M2 polarization through exosome PD-L1, which weakened the anti-PD-1/PD-L1 curative effect, and finally led to anti-PD-1/PD-L1 treatment resistance (Liu et al., 2021).

In fact, it is not difficult to see that the expression of PD-L1 is upregulated in different tumor cells. The important mechanism of tumor immune escape is the combination of PD-L1 with PD-1 of T cells, which causes an immune checkpoint response. With the increasing number of studies on exosome PD-L1 recently, its role in research has become more and more important. Related studies have found that the exosome PD-L1 and cell surface PD-L1 have the same membrane topology by using enzyme-linked immunosorbent assay (Chen et al., 2018). Exosomes containing PD-L1 secreted by tumors can effectively transfer exosomes PD-L1 to macrophages and weaken anti-tumor immunity in tumor microenvironment (Yang et al., 2018). However, the mechanism of how exosome PD-L1 induces immunosuppression has not yet been fully elucidated. Liu et al. (2021) confirmed that by increasing the expression of melanoma exosomes PD-L1, the M2 polarization of macrophages can be induced, which ultimately leads to resistance to anti-PD-1/PD-L1 treatment. In addition, this method is consistent with the results of tumor immune escape and anti-PD-L1 treatment caused by directly upregulating PD-L1 in macrophages (Zhang et al., 2017; Wen et al., 2018). All these indicate that tumor-derived exosomes PD-L1 can promote tumor immune escape by inducing the polarization of macrophages M2.

## Immunosuppressive Effects of Tumor-Derived Exosome PD-L1 on Distinct Cancers

### Non-small Cell Lung Cancer

Lung cancer is the leading cause of cancer-related death worldwide (Siegel et al., 2012). Various targeted therapies and immunotherapies for NSCLC have been gradually and effectively applied (He et al., 2015; Grigg and Rizvi, 2016). Among them, PD-1/PD-L1 inhibitors are representative and have improved the clinical efficacy of NSCLC treatment to a certain extent (Herbst et al., 2016; Brahmer et al., 2017; Mok et al., 2019). Measurement of the expression level of exosome PD-L1 plays a fundamental role in the diagnosis and prognosis of NSCLC. However, there is still no expected response of NSCLC patients with positive immunohistochemical staining for PD-L1 to immunotherapy, and the reason remains elusive. Li et al. (2019) showed that exosome PD-L1 levels were significantly higher in NSCLC patients (especially in advanced-stage individuals) than in healthy controls. The level of exosome PD-L1 was obviously related to tumor size, positive lymph node status, distant metastasis and TNM stage (Li et al., 2019). Kim et al. (2019) found that exosomes containing PD-L1 could be isolated from the plasma of patients with NSCLC. Exosome PD-L1 plays an important role in tumor immune escape by inhibiting cytokines and inducing CD8 T cell apoptosis. Liu et al. measured exosome PD-L1 expression in NSCLC patients using a compact surface plasmon resonance (SPR) biosensor and found that exosome PD-L1 expression was significantly higher than that in healthy controls (Liu et al., 2018). Exosome PD-L1 from NSCLC cells has also been shown to mediate immune escape by inhibiting cytokines (IL-2 and IFN-γ) and inducing CD8 T cell apoptosis (Kim et al., 2019). In short, exosome PD-L1 may be a novel biomarker and a promising target for lung cancer.

### Melanoma

Melanoma is a typical immunosuppressive malignant tumor with a high possibility of distant metastasis (Aubuchon et al., 2017). At present, immune checkpoint inhibitor therapy targeting PD-L1 has made remarkable achievements in the treatment of melanoma (Chen and Han, 2015; Topalian et al., 2016). However, the currently approved response rate of patients with advanced melanoma to monoclonal antibodies is still not satisfactory (Ribas et al., 2016; Zaretsky et al., 2016). Therefore, the identification of a typical biomarker is critical for the diagnosis and treatment of melanoma. The original intention was to focus on PD-L1 expression in tumors and blood samples; however, these test results were not necessarily reliable due to the inhibition of PD-L1 in tumors and the instability of PD-L1 in blood. Recently, exosome PD-L1 has been identified as a potential biomarker of melanoma. Cordonnier et al. (2020) further confirmed that circulating exosome PD-L1 in melanoma patients plays a role through T lymphocytes in secondary lymphoid organs and through the immunosuppressive PD-1/PD-L1 pathway. Moreover, a large increase in exosome PD-L1 is related to tumor progression (Cordonnier et al., 2020). Chen et al. (2018) demonstrated the presence of melanoma-associated exosome PD-L1 and its immunosuppressive effects and suggested that the exosome PD-L1 level is an indicator to distinguish clinical responders from non-responders.

### Gastric Cancer

Gastric cancer is the fourth most common cancer in the world (Xiang et al., 2016). The efficacy of anti-PD-1 therapy in metastatic gastric cancer seems to be quite promising (Muro et al., 2016). A recent study have shown that exosome PD-L1 is required to predict the prognosis of gastric cancer patients. Fan et al. (2019) showed that there was a significant correlation between the level of exosome PD-L1 and the stage of gastric cancer, and the survival rate was worse in the group with higher exosome PD-L1 expression. The OS of patients with high exosome PD-L1 expression was significantly lower than that of patients in the low expression group with both stages I and II AJCC, demonstrating the predictive value of exosome PD-L1 for the OS of patients with early gastric cancer.

### Breast Cancer

HER2 expression is elevated in 25% of breast cancer patients and is often accompanied by a poor prognosis (Slamon et al., 1989). At present, breast cancer treatments targeting HER2 have achieved some efficacy in clinical practice. However, not all breast cancer patients who overexpress HER2 respond to therapy, and many patients still develop treatment resistance (Nahta and Esteva, 2007). Notably, enhanced drug resistance is likely to cause the immune escape of cancer cells (Bruttel and Wischhusen, 2014). Chatterjee et al. (2020) reported that breast cancer cells secrete exosomes carrying PD-L1 and are highly immunosuppressive. Additionally, exosome PD-L1 is stimulated by TGF-β, which blocks the phosphorylation of src family proteins and promotes CD8 T cell dysfunction. Therefore, exosome PD-L1 has considerable potential for the diagnosis and treatment of breast cancer patients (Martinez et al., 2017).

### Pancreatic Cancer

Pancreatic cancer is one of the cancers with the highest mortality rate, and the detection of exosome PD-L1 in the blood is a good prognostic indicator of pancreatic cancer. Pancreatic ductal adenocarcinoma (PDAC) is the most common histological subtype of malignant pancreatic cancer (Rahbari et al., 2016), accounting for 90% of all cases. Because this type of malignant tumor is highly invasive and infiltrative, most diagnoses are made at the advanced tumor stage. The presence of a tumor immune escape mechanism leads to the rapid development of pancreatic cancer. To date, patients with pancreatic cancer have hardly responded to monotherapy with checkpoint inhibitors (Foley et al., 2016). PD-L1 is highly expressed in pancreatic cancer and is associated with poor prognosis (Geng et al., 2008; Chen et al., 2009; Wang et al., 2010). Therefore, the expression of tumor-derived exosome PD-L1 will greatly improve the diagnostic status of pancreatic cancer and immunotherapy. Lux et al. (2019) examined the expression of PD-L1 in blood samples and showed that the survival time of PD-L1-positive patients was significantly lower than that of PD-L1-negative patients. Therefore, the expression of PD-L1 in exosomes has profound significance for the prognosis of pancreatic cancer. However, since exosome PD-L1 expression in CP patients is higher than that in PDAC patients, exosome PD-L1 may not be suitable as a diagnostic indicator for pancreatic cancer (Lux et al., 2019).

### Head and Neck Squamous Cell Carcinoma

Head and neck squamous cell carcinoma is a common and lethal disease with the highest diagnostic rate in the world (Ferlay et al., 2013; Mourad et al., 2017). The tumor microenvironment of HNSCC has strong immunosuppressive properties, and HNSCC is a highly immunosuppressive, malignant tumor (Ferris et al., 2006; Bergmann et al., 2007; Whiteside, 2018). The PD-1/PD-L1 immunosuppressive pathway has received great attention. A series of experimental studies showed that patients with excessive plasma exosome PD-L1 had higher disease activity than patients with lower exosome PD-L1 levels. Higher plasma levels of exosome PD-L1 were associated with stronger inhibition of CD8 effector T cell activation, and anti-PD-1 Abs significantly reduced the dose-dependent effect of exosome PD-L1 on T cell activity and sex-associated inhibition (Theodoraki et al., 2018). Therefore, in HNSCC, exosome PD-L1 binds to the PD-1 receptor on the surface of activated T cells to maintain their biological activity and effectively transmit signals, thereby affecting the function of immune cells and leading to immune escape. The level of tumor-derived exosomes can be used as an indicator to reflect the efficacy of patient treatment (Theodoraki et al., 2019). Theodoraki et al. (2018) isolated exosomes carrying PD-L1 from the plasma of HNSCC patients and inhibiting T cell function, demonstrating that circulating exosome PD-L1 may be a useful indicator of disease and immune activity in HNSCC patients.

### Lymphoma

Lymphoma is a primary malignant tumor of lymph nodes or lymph tissues, and its occurrence may be related to gene mutations (Zahn et al., 2020), virus and other pathogen infections (Huang et al., 2021). Studies highlight the roles of clonally diverse CD4 T cells and innate effectors in the efficacy of PD-1 blockade in classical Hodgkin lymphomas (Cader et al., 2020). Additionally, study show that diffuse large B-cell lymphomas possess a self-organized infrastructure comprising side population (SP) and non-SP cells, where transitions between clonogenic states are modulated by exosome-mediated WNT signaling (Koch et al., 2014). Li et al. evaluated the prognostic value of pretreatment circulating sPD-L1 and exoPD-L1 in extranodal NK/T cell lymphoma patients (Li et al., 2020). Their study revealed that circulating exoPD-L1 and sPD-L1 levels were significantly elevated in extranodal NK/T cell lymphoma and might be promising biomarkers for evaluating the survival outcomes of extranodal NK/T cell lymphoma patients.

## Future Clinical Applications of Exosome PD-L1

### Development of Exosome PD-L1 Detection Methods

Although the application of immunotherapy has shown considerable value in the diagnosis, treatment, and prediction of various cancers, the response rates of patients with positive PD-L1 pathology to immunotherapy is only 10–30% (Fehrenbacher et al., 2016; Eggermont et al., 2018). The level of exosome PD-L1 can reflect the occurrence of tumors in certain cancer types and has a strong correlation with the response to immunotherapy (Chen et al., 2018). Therefore, the detection of exosome PD-L1 levels can be used as a supplement to existing immune checkpoint measurements to increase the accuracy of diagnosis. At present, the accepted quantitative detection method for exosome PD-L1 is enzyme-linked immunosorbent assay (ELISA; Welton et al., 2010; Nawaz et al., 2014; Chen et al., 2018). However, this method has certain limitations. When exosome PD-L1 expression is too low (<200 pg/ml), it is impossible to distinguish between patients and healthy people (Ramirez et al., 2018). Furthermore, Huang et al. (2020) proposed a detection method called HOLMES-ExoPD-L1 that replaces ELISA to quantitatively detect exosome PD-L1. By applying an aptamer with a higher recognition efficiency than the PD-L1 antibody, the detection sensitivity is significantly improved. The uniformity of thermophoresis is used to promote faster binding of the aptamer to exosome PD-L1 (Huang et al., 2020). Pang et al. (2020) proposed an *in vitro* assay to detect plasma exosome PD-L1, which is undetectable by ELISA. The principle involves the use of nanoparticles to enrich exosomes by binding the TiO_2_ shell and the hydrophilic phosphate head of exosome phospholipids. This method efficiently captures up to 96.5% of exosomes, which are then quantified by labeling exosome PD-L1 with a specifically labeled anti-PD-L1 antibody (Pang et al., 2020). Liu et al. (2018) developed a compact surface plasmon resonance (SPR) biosensor with the same principle as traditional SPR, which is a highly sensitive, real-time, label-free optical detection method that does not require nanomaterials and effectively reduces the detection cost. Researchers analyzed NSCLC serum samples with this method and found that the expression of exosome PD-L1 in patients with NSCLC was increased. Surprisingly, this method has a higher detection sensitivity than the traditional ELISA detection method. With the same sample size, the researchers used this method to detect exosome PD-L1 levels that ELISA could not detect (Liu et al., 2018; Table 2).

**TABLE 2:** Exosome PD-L1 detection method.

**Method**	**Mechanism**	**Advantage**	**Disadvantages**
Enzyme-linked immunosorbent assay (ELISA)	The PD-L1 antigen and antibody are adsorbed on the surface of the solid phase carrier, allowing the antigen and antibody to react on the surface	Strong specificity, Fast Low cost	Low sensitivity, When the expression of PD-L1 in exosomes is too low (<200 pg/ml), it is impossible to distinguish patients from healthy people
HOLMES-ExoPD-L1	Due to the different depletion rates, the extracellular aptamers can observe strong fluorescence	Compared with PD-L1 antibody, the use of aptamers provides higher recognition efficiency, which can significantly improve the detection sensitivity, The operation is simple	The biological stability of aptamers is poor compared to antibodies, and the short half-life *in vivo* limits the development of aptamers in clinical applications
Based on Fe_3_O_4_@TiO_2_ isolation and SERS immunoassay	Fe_3_O_4_@TiO_2_ nanoparticles are used to enrich exosomes by combining the TiO_2_ shell with the hydrophilic phosphate head of exosome phospholipids, followed by the addition of Au @ Ag @ MBA SERS tag modified with anti-PD-L1 antibody to mark the outside exosome PD-L1 for quantification	The speed is faster, Exosome PD-L1 can be captured and analyzed directly from the serum	With the use of nanomaterials, the cost may be higher
Compact surface plasmon resonance (SPR) biosensor	The same as the traditional SPR sensing mechanism	High-sensitivity, label-free, real-time optical detection method	Need to use its special equipment, there is a certain learning cost

### Early Diagnosis and Prognosis of Cancer

Surgery is still the preferred method for radical treatment of tumors, but quite a lot of cancer patients are usually diagnosed at the advanced stage, thus missing the best opportunity for treatment. For example, most patients with gastric cancer are usually diagnosed at the advanced stage, and the 5-year survival rate is less than 20% (Price et al., 2012). 75% of lung cancer patients are already in the advanced stage when they are discovered (Steinman and Banchereau, 2007). Pancreatic ductal adenocarcinoma (PDAC) is highly aggressive and invasive, most of the diagnoses are performed in the advanced tumor stage (Rahbari et al., 2016). Therefore, exploring reliable indicators for early cancer diagnosis and prognostic factors has far-reaching significance for cancer diagnosis and treatment. Many reports show that PD-L1 is abnormally highly expressed in a variety of tumors (skin, brain, thyroid, esophagus, colorectal, etc.) (Iwai et al., 2002; Taube et al., 2014; Patel and Kurzrock, 2015). However, due to the inhibition of PD-L1 in tumors and the instability of PD-L1 in blood samples, some studies have shown that there is no difference in the concentration of sPD-L1 between NSCLC patients and healthy blood donors (Li et al., 2019). Therefore, simply detecting PD-L1 in tumors or blood is very unreliable for the early diagnosis of tumors. We know that exosomes have been widely regarded as a new type of crosstalk circuit between tumor cells and the tumor microenvironment (Li et al., 2015; Melo et al., 2015; Tang and Wong, 2015). Some studies have clarified that exosomes even represent the mechanism by which immunosuppressive agents in TME participate in the tumor progression cycle (Whiteside, 2016; Ludwig et al., 2017). Many current studies have shown that the detection of the expression level of exosomes PD-L1 is of great significance for the early diagnosis of tumors (Chen et al., 2018; Li et al., 2019). Li et al. (2019) showed that the level of exosome PD-L1 in NSCLC patients (especially advanced patients) was significantly higher than that in healthy controls. The level of exosome PD-L1 was significantly correlated with tumor size, lymph node positive status, distant metastasis and TNM stage (Li et al., 2019). However, the level of sPD-L1 is not related to clinicopathological features other than tumor size. Liu et al. (2018) measured the expression level of exosome PD-L1 in NSCLC patients by using a compact surface plasmon resonance (SPR) biosensor and found that the expression of exosome PD-L1 was significantly higher than that in healthy controls. The above studies show that exosome PD-L1 may become a promising biomarker for the diagnosis of lung cancer. In the plasma of melanoma patients, the level of exosome PD-L1 was significantly higher than that of sPD-L1, and exosome PD-L1 was detected in all patients. Although the level of exosome PD-L1 has no relationship to clinicopathological features, the change after treatment (ΔExoPD-L1) is related to tumor response to treatment, and it is verified that the increase of exosome PD-L1 is related to tumor progression (Cordonnier et al., 2020). Fan et al. (2019) showed that there was a significant correlation between the level of exosome PD-L1 and the stage of gastric cancer, and the survival rate was worse in the group with higher exosome PD-L1 expression. The OS of patients with high exosome PD-L1 expression was significantly lower than that of patients in the low expression group with both stages I and II AJCC, demonstrating the predictive value of exosome PD-L1 for the OS of patients with early gastric cancer (Fan et al., 2019). The above-mentioned studies show that the exosome PD-L1 is more reliable than tumor and serum PD-L1, and it is of great significance for the early diagnosis and prognosis of cancer.

### Exosome PD-L1 as a Biomarker for Clinical Anti-PD-1/PD-L1 Therapy

**FIGURE 2:**
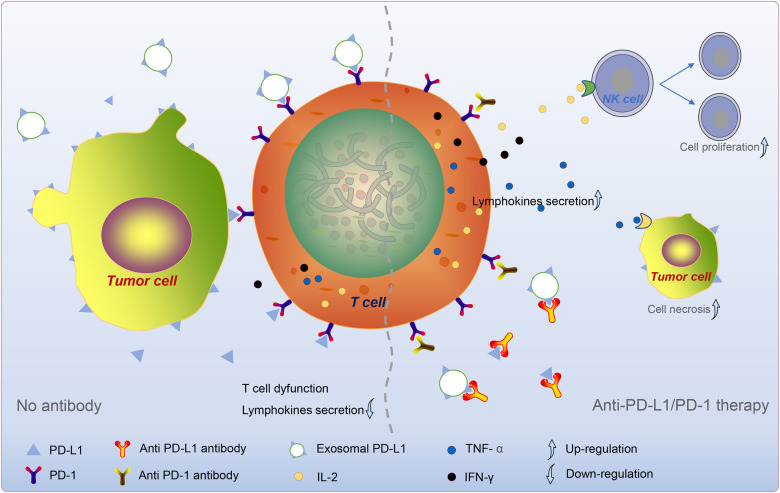
Tumor cell-derived exosome PD-L1-mediated immune escape. Exosome PD-L1, which synergizes with sPD-L1 and PD-L1 on the surface of tumor cells, binds to PD-1 on T cells and reduces T cell activity and lymphokine secretion. In anti-PD-L1/PD-1 therapy, the antibody specifically binds to PD-L1/PD-1, prevents the coupling of circulating PD-L1 to PD-1, increases the secretion of cytokines. Subsequently, these cytokines, such as IL-2, TNF-α, and IFN-γ, could give rise to the division and proliferation of NK cells and the death of tumor cells. The dotted line divides the picture into left and right sides. The left part mainly describes the “No antibodies” situation, and the right part mainly talks about the “anti-pd-1/PD-L1 therapy” situation.

Programmed death ligand 1 is rarely expressed on the cell surface of normal human tissues and is abundantly expressed on the surface of cancer cells (Dong et al., 2002). Additionally, IFN-γ upregulates PD-L1 on the surface of normal tissue cells and cancer cells. The use of an anti-human PD-L1 antibody prevents the effect of tumor cell PD-L1 on activated effector T cells and blocks the interaction of PD-L1 with T cells. This finding shows that the use of an anti-PD-L1 antibody inhibits the progressive growth of mouse tumors (Dong et al., 2002). PD-L1 inhibits the anti-tumor function of T cells by activating PD-1. The PD-L1 signaling pathway causes immune escape of tumor cells in the tumor microenvironment (TME; Mittal et al., 2014). Subsequent studies confirmed the accuracy of this concept (Iwai et al., 2002; Strome et al., 2003). These studies showed that tumors evade immune attack through the PD-1/PD-L1 pathway and provide an anti-PD-1/PD-L1 approach for cancer therapy (Figure 2).

Additionally, the FDA has approved two PD-1 antibodies for the treatment of human cancer. Multiple clinical studies have shown that anti-PD-1/PD-L1 therapy has exceedingly significant clinical importance for improving the survival rates of patients with advanced and metastatic tumors. Moreover, in a variety of cancers, especially solid tumors, anti-PD-1/PD-L1 therapy has a fairly long-lasting effect. Notably, the removal of exosome PD-L1 inhibits tumor growth, even in models that are resistant to anti-PD-L1 antibodies (Poggio et al., 2019). In some studies of metastatic melanoma, anti-PD-1/PD-L1 therapy showed profound application prospects (Ribas et al., 2016; Topalian et al., 2016). At present, immunohistochemical (IHC) staining of PD-L1 is routinely tested in clinical practice to predict the effect of anti-PD-1/PD-L1 immunotherapy (Martinez and Gordon, 2014). Compared with the PD-L1 negative/weak expression group, the remission rate of the PD-L1 high expression group increased from 8 to 30% (Funes et al., 2018). But even so, a considerable number of NSCLC patients with positive PD-L1 IHC staining have unsatisfactory immunotherapy effects (Gabrusiewicz et al., 2018). Among them, PD-1/PD-L1 inhibitors are represented, which have improved the clinical efficacy of NSCLC and other tumors to a certain extent (Okazaki et al., 2017; Chen et al., 2018; Liu et al., 2021). However, many tumor patients with positive immunohistochemical staining for PD-L1 still have no expected response after receiving immunotherapy.

In gastric cancer patients, not all PD-L1 positive patients respond to anti-PD-1, and even PD-L1-negative patients respond (Wen et al., 2018). The reason for this disappointing result is not yet clear, but it is likely that the integrated mechanism of the PD-L1 pathway in TME is not fully understood. Therefore, we need to have a deeper understanding of the immunosuppressive pathway of PD-1/PD-L1 to better improve the treatment efficacy in patients. Recent studies have shown that circulating exosome PD-L1 promotes activated T cell apoptosis and inhibits the production of cytokines (Chen et al., 2018). Antibodies against PD-L1 or PD-1 block the inhibitory effect of exosome PD-L1 on T cells. Exosome PD-L1 may reflect the dynamic interaction between tumors and immune cells (Chen et al., 2018). In treated patients, the recovery of T cell activity was negatively correlated with an increase in exosome PD-L1. Exosome PD-L1 reflected whether anti-PD-1 therapy successfully triggered anti-tumor immunity. Currently, circulating exosome PD-L1 has been used as a predictive biomarker of the clinical response to anti-PD-1 therapy. However, given that the dynamic expression of tumor PD-L1 is lower than that of exosome PD-L1 and the detection of tumor PD-L1 requires invasive tumor biopsies, exosome PD-L1 may be a promising blood-based biomarker. Subsequent confirmation of the clinical application potential of exosome PD-L1 in multiple cancer types is required. For example, Theodoraki et al. (2018) showed that in HNSCC patients, higher plasma levels of exosome PD-L1 were associated with stronger inhibition of CD8 effector T cell activation. Anti-PD-1 Abs significantly reduced the dose-dependent inhibition of T cell activity by exosome PD-L1 (Theodoraki et al., 2019). Poggio et al. (2019) found that exosome PD-L1 seems to be resistant to anti-PD-L1 treatment. At the same time, inhibiting exosome PD-L1 helps maintain long-lasting anti-tumor immunity (Zhang et al., 2017). A study by Del Re et al. (2018) explored the relationship between exosome PD-L1 mRNA expression and response to anti-PD-1 treatment in melanoma (*n* = 18) and non-small cell lung cancer (*n* = 8). They emphasized that exosome PD-L1 should be considered when predicting the outcome of anti-PD-1 treatment (Siegel et al., 2012). This may also be the reason why the PD-L1 IHC profile of the tumor is not an ideal biomarker for the selection of anti-PD-1/PD-L1 immunotherapy candidates. Based on the above conclusions, we know that we cannot simply detect the tumor PD-L1 for verification. But if there is a serological marker that can provide reliable information on the expression status of tumor PD-L1, this situation can be greatly improved. The exosome PD-L1 is very likely to be this serological marker.

## Conclusion

Tumor-derived exosomes PD-L1 play a key role in tumor immune escape of tumors. At present, there are several mechanisms by which cell surface or exosome PD-L1 mediates tumor immunity to achieve immune escape, such as by inducing activated T cell apoptosis, promoting T cell weakness, enhancing the function of Tregs, inhibiting T cell proliferation, and inhibiting impaired T cell activation and IL-2 production.

Many studies have confirmed that PD-L1 on tumor cells mediates immunosuppressive effects. Similarly, PD-L1 secreted by tumor cells on the surface of exosomes binds to PD-1 on T cells and exerts biological effects. However, further exploration of the molecular mechanisms is needed. Exosome PD-L1 has been shown to be of clinical value in the diagnosis, treatment and prognosis of various cancers, such as NSCLC, melanoma, gastric cancer, breast cancer and HNSCC. The measurement of PD-L1 levels in exosomes complements existing immune checkpoint measurements, facilitating the accuracy of immune-related tumor diagnosis. In addition, exosome PD-L1 contributes to anti-PD-L1/PD-1 therapy and enhances the sensitivity of tumor patients to treatment. Nevertheless, more work remains to be done to apply tumor-derived exosome PD-L1 in clinical practice.

## Author Contributions

ZS provided direction and guidance throughout the preparation of this manuscript. BS and QD wrote and edited the manuscript. ZC, CC, QZ, and SH reviewed and made significant revisions of the manuscript. BQ, JL, GW, and WY collected and prepared the related manuscript. All authors read and approved the final manuscript.

## Conflict of Interest

The authors declare that the research was conducted in the absence of any commercial or financial relationships that could be construed as a potential conflict of interest.

## Publisher’s Note

All claims expressed in this article are solely those of the authors and do not necessarily represent those of their affiliated organizations, or those of the publisher, the editors and the reviewers. Any product that may be evaluated in this article, or claim that may be made by its manufacturer, is not guaranteed or endorsed by the publisher.

## Abbreviations

PD-L1: programmed death ligand 1
PD-1: programmed cell death 1
ILV: intraluminal vesicles
MVE: multiple vesicle endosomes
ESCRT: endosomal complex required for transportation
TAM: tumor-associated macrophages
sPD-L1: soluble PD-L1
DCs: dendritic cells
Tregs: regulatory T cells
NSCLC: non-small cell lung cancer
HNSCC: Head and neck squamous cell carcinoma
PD-L1 (KD) B16-F10: knockout of PD-L1-expressed B16-F10 cells
APCs: antigen presenting cells
CTL: cytotoxic T lymphocytes
HSCs: hematopoietic stem cells
SPR: surface plasmon resonance
PDAC: Pancreatic ductal adenocarcinoma
ELISA: enzyme-linked immunosorbent assay.
